# Exposure of Mesenchymal Stem Cells to an Alzheimer's Disease Environment Enhances Therapeutic Effects

**DOI:** 10.1155/2021/6660186

**Published:** 2021-03-16

**Authors:** Sang Eon Park, Hyeong Seop Kim, Soo Jin Kwon, Min-Jeong Kim, Suk-joo Choi, Soo-young Oh, Gyu Ha Ryu, Hong Bae Jeon, Duk L. Na, Jong Wook Chang

**Affiliations:** ^1^Stem Cell Institute, ENCell Co. Ltd, Seoul 06072, Republic of Korea; ^2^Stem Cell & Regenerative Medicine Institute, Samsung Medical Center, Seoul 06351, Republic of Korea; ^3^Department of Obstetrics and Gynecology, Samsung Medical Center, Seoul 06351, Republic of Korea; ^4^Department of Medical Device Management and Research, SAIHST, Sungkyunkwan University School of Medicine, Seoul 06351, Republic of Korea; ^5^The Office of R&D Strategy & Planning, Samsung Medical Center, Seoul 06351, Republic of Korea; ^6^Department of Neurology, Samsung Medical Center, Sungkyunkwan University School of Medicine, 81 Irwon-ro, Gangnam-gu, Seoul 06351, Republic of Korea; ^7^Samsung Alzheimer Research Center, Samsung Medical Center, Seoul 06351, Republic of Korea

## Abstract

Mesenchymal stem cells (MSCs) have emerged as a promising tool for the treatment of Alzheimer's disease (AD). Previous studies suggested that the coculture of human MSCs with AD in an *in vitro* model reduced the expression of amyloid-beta 42 (A*β*42) in the medium as well as the overexpression of amyloid-beta- (A*β*-) degrading enzymes such as neprilysin (NEP). We focused on the role of primed MSCs (human Wharton's jelly-derived mesenchymal stem cells (WJ-MSCs) exposed to an AD cell line via a coculture system) in reducing the levels of A*β* and inhibiting cell death. We demonstrated that mouse groups treated with naïve MSCs and primed MSCs showed significant reductions in cell death, ubiquitin conjugate levels, and A*β* levels, but the effects were greater in primed MSCs. Also, mRNA sequencing data analysis indicated that high levels of TGF-*β* induced primed-MSCs. Furthermore, treatment with TGF-*β* reduced A*β* expression in an AD transgenic mouse model. These results highlighted AD environmental preconditioning is a promising strategy to reduce cell death and ubiquitin conjugate levels and maintain the stemness of MSCs. Further, these data suggest that human WJ-MSCs exposed to an AD environment may represent a promising and novel therapy for AD.

## 1. Introduction

Alzheimer's disease (AD) is a widespread cause of dementia and is an age-related [1, 2], progressive, and irreversible neurodegenerative disease [3, 4] for which no disease-modifying therapy exists [5, 6]. Most of the drugs being developed target A*β* alone [7, 8]. The development of a multitarget drug, however, may be more effective given the multiple pathogenic mechanisms involved in AD [9, 10].

Prior studies including those reported by our group suggest that mesenchymal stem cells (MSCs) may be a potential treatment for AD [11–16]. MSCs secrete proteins that inhibit apoptosis and inflammation, modulate the immune response in damaged tissues, and promote endogenous neurogenesis and neuroprotection. Based on the specific mechanisms induced and the improved therapeutic outcomes, MSCs show considerable promise [17]. When used to treat AD, MSCs expressed genes related to enhanced extracellular transport and secretion [11–13, 15, 16], which indicates an increase in paracrine activity. These genes are known to exhibit neuroprotective and neurotrophic features such as the inhibition of apoptosis, the regulation of cell proliferation, and the regulation of neurogenesis. Further, our previous study demonstrated that MSCs exposed to cerebrospinal fluid (CSF) of AD patients upregulated the genes related to AD treatment while maintaining stemness [18]. Therefore, AD-exposed MSCs enhanced the overall efficacy of MSCs in AD therapy.

In this study, we investigated whether the therapeutic potency of MSCs could be enhanced by exposing them to an AD environment. Therefore, we generated AD-exposed MSCs using a coculture of MSCs and the APP695-Swedish mutant (K595N/M596L)-expressing H4 cell (H4SW cell) line, which provided an AD environment characterized by high levels of secreted toxic forms of A*β*, such as A*β*1–40 and A*β*1–42 [19, 20]. We then analyzed the therapeutic effects of the MSCs following exposure to the AD environment. Furthermore, to identify the genes expressed by conditioned MSCs, which were therapeutically effective in AD, we performed mRNA sequencing analysis of both the naïve and conditioned MSCs.

## 2. Materials and Methods

### 2.1. Wharton's Jelly-Derived Mesenchymal Stem Cell Culture

Wharton's jelly-derived mesenchymal stem cells (WJ-MSCs) were isolated according to the procedure described by Kwon et al. [21]. The WJ-MSCs were cultured according to the standard operating procedures (SOPs) of the Good Manufacturing Practice facility at Samsung Medical Center. Prior to coculturing with H4SW cells, the WJ-MSCs were detached using a 0.25% trypsin-EDTA solution (Gibco-Invitrogen).

### 2.2. H4 and H4SW Cell Line Culture

Human glioblastoma H4 cells and APP695-Swedish mutant (K595N/M596L)-expressing H4 cells (H4SW) were kindly provided by Jung-Hyuck Ahn's lab (Ewha Woman's University School of Medicine, Republic of Korea) and cultured according to the procedure reported previously [19, 20]. H4 and H4SW cells were cultured in Dulbecco's modified Eagle's media (DMEM; Gibco/BRL) containing 10% fetal bovine serum (FBS; Gibco/BRL), 100 U/mL penicillin (Gibco/BRL), 100 *μ*g/mL streptomycin (Gibco/BRL), and 2 mM L-glutamine (Gibco/BRL) as described previously. The H4SW cell cultures were maintained by adding 500 *μ*g/mL geneticin (Gibco/BRL) to the growth media.

### 2.3. Preconditioning MSCs under an AD Environment

H4SW cells were cultured and maintained on a 6-well plate compatible with insert wells. Upon reaching 70% confluency, the H4SW cells were cocultured with 1 × 10^5^ WJ-MSCs on 6-well transwell inserts (BD Falcon, USA) for 24 h in a serum-free medium at 37°C with 5% CO_2_.

### 2.4. Flow-Cytometry Analysis for Validating Reconditioned WJ-MSCs

After coculture, the preconditioned WJ-MSCs at passage five were detached using a 0.25% trypsin-EDTA solution and harvested in a 15 mL conical tube. After centrifugation, the WJ-MSCs were washed and resuspended in phosphate-buffered saline (PBS) with 2% FBS to block nonspecific binding sites. Immunophenotypic analysis of the preconditioned WJ-MSCs was performed according to the MSC criteria of the International Society for Cell Therapy (ISCT) [22] via flow cytometry to determine the expression of the following markers: CD44, CD73, CD90, CD105, CD14, CD11b, HLA-DR (MHC-II), CD34, CD45, and CD19 (BD Biosciences, USA). At least 10,000 events were acquired on a BD FACSVerse (BD Biosciences, NJ, USA), and the results were analyzed with BD FACSuite software version 10 (BD Biosciences, USA). The differentiation of preconditioned WJ-MSCs was analyzed according to the procedure outlined in a previous report [21].

### 2.5. H4SW Cell Coculture with Preconditioned WJ-MSCs

At 70% confluency, H4SW cells (in the lower chamber of the Transwell unit) were cocultured with 1 × 10^5^ preconditioned WJ-MSCs seeded on 6-well transwell inserts (BD Falcon) for 24 h under serum-free conditions at 37°C with 5% CO_2_. Naive WJ-MSCs were cocultured with H4SW cells as a control group. After coculture for 24 h, the H4SW cells were harvested and rapidly frozen for further analysis.

### 2.6. Intraventricular Injection of WJ-MSCs and TGF-*β* into 5XFAD Mice

A 12-month-old transgenic mouse model of AD, 5xFAD (MMRC #04848), was used in this study. The mice were purchased from the Jackson Laboratory (Bar Harbor, ME, USA). Experimental animals were divided into five groups: wild-type (WT), 5xFAD (sham), +naïve MSC (naïve MSCs were injected into 5xFAD mice), +primed MSCs (primed MSCs were injected into 5xFAD mice), and +TGF-*β* (recombinant TGF-*β* proteins were injected into 5xFAD mice). Before injecting WJ-MSCs, all the mice were anesthetized and maintained on 5% isoflurane with 2% isoflurane inhalation during the surgical procedure. After shaving and sterilizing the surgical site with povidone-iodine, a skin incision of approximately 1 cm in length was made. Using a microdrill, a small burr hole was made at the following coordinates (right lateral ventricle): A/P-0.4 mm, M/L+1.0 mm, and D/V-2.3 mm from the bregma. WJ-MSCs (1 × 10^5^ cells) suspended in 3 *μ*L of phenol-red MEM-alpha or 3 *μ*L of TGF-*β* (10 ng/mL) were injected into the right lateral ventricle at a rate of 1 *μ*L per min with a 15 min delay using a Hamilton syringe (Hamilton Company, NV, USA). The needle was carefully removed after the injection was completed, and the skin was sutured, followed by sterilization of the area. All mice were euthanized one week after administration.

### 2.7. Brain Tissue Preparation

One week after the injection of WJ-MSCs, all mice were anesthetized with isoflurane, followed by cardiac perfusion. The brain tissue from the mice was harvested and divided in half along the longitudinal fissure. The harvested brain tissues were frozen in liquid nitrogen and stored at -80°C for Western blots and enzyme-linked immunosorbent assay (ELISA) analysis or fixed in 4% paraformaldehyde for histological analysis.

### 2.8. Western Blots and ELISA

Tissue and cell extracts were prepared according to previously published methods [23]. Briefly, ultrasonication (Branson Ultrasonics, Slough, UK) was performed in a buffer containing 9.8 M urea, 4% 3-((3-cholamidopropyl) dimethylammonio)-1-propanesulfonate (CHAPS), 130 mM dithiothreitol, 40 mM tris-Cl, and 0.1% sodium dodecyl sulfate (SDS). The protein concentrations were measured using the Bradford assay (Bio-Rad Laboratories, Inc., CA, USA). Protein extracts (20 *μ*g/lane) were loaded onto SDS-polyacrylamide gels for electrophoresis and then transferred to nitrocellulose membranes. NuPAGE 12% (Invitrogen, CA, USA) gels were used for the immunoblot analysis. The membranes were incubated with anti-ubiquitin antibodies (Ub, 1 : 1,000; Santa Cruz, USA), anti-MOAB (1 : 200; Novus Biologicals, CO, USA), anti-GFAP (1 : 1,000; Abcam, Cambridge, UK), anti-TNF-*α* (1 : 5,000; Enogene, Nanjing, China), and anti-*β*-actin (1 : 5,000; Sigma-Aldrich Co., MO, USA) at 4°C overnight. Subsequently, the membranes were incubated with a secondary antibody (goat anti-mouse IgG-HRP; Ab Frontier, USA) for 1 h at room temperature (RT). The blots were developed using ECL solution (Advansta, USA), and the protein bands were detected via exposure to X-ray film. Densitometric analysis was performed using ImageJ software (NIH, USA).

The ELISA tests were performed with an A*β*42 ELISA kit (Wako, Cambridge, UK) and an SRGN ELISA kit (LifeSpan BioSciences, Washington, USA) according to the manufacturers' instructions.

### 2.9. Thioflavin-S Staining

Fixed brain tissues were embedded in paraffin, and 4 *μ*m thick coronal sections were prepared. To detect A*β*, thioflavin-S staining was performed according to the manufacturer's instructions. All slides were deparaffinized by serial hydration using a graded ethanol series, followed by treatment of the slides with 1% filtered thioflavin-S (Sigma-Aldrich) and washing. The mounted slides were stored at 4°C before fluorescence microscopy imaging (Nikon, Shinagawa, Tokyo, Japan).

### 2.10. RNA Isolation

Total RNA was isolated using TRIzol reagent (Invitrogen). The RNA quality was assessed by an Agilent 2100 bioanalyzer using the RNA 6000 Nano Chip (Agilent Technologies, Amstelveen, The Netherlands), and RNA quantification was performed using an ND-2000 Spectrophotometer (Thermo Inc., DE, USA).

### 2.11. Library Preparation and QuantSeq 3′ mRNA Sequencing

Libraries were constructed from the control and test RNAs using a QuantSeq 3′ mRNA-Seq Library Prep Kit (Lexogen, Inc., Austria) according to the manufacturer's instructions. In brief, 500 ng of each total RNA sample was prepared and an oligo-dT primer containing an Illumina-compatible sequence at its 5′ end was hybridized to the RNA, and reverse transcription was performed. After degradation of the RNA template, the second-strand synthesis was initiated by a random primer containing an Illumina-compatible linker sequence at its 5′ end. The double-stranded library was purified by magnetic beads to remove all reaction components. The library was amplified to add the complete adapter sequences required for cluster generation. The finished library was purified from the PCR components. High-throughput sequencing was performed via single-end 75 sequencing using NextSeq 500 (Illumina, Inc., USA).

### 2.12. QuantSeq 3′ mRNA Sequencing Data Analysis

The QuantSeq 3′ mRNA-Seq reads were aligned using Bowtie2 [24]. Bowtie2 indices were either generated from the genome assembly sequence or the representative transcript sequences for aligning with the genome and transcriptome. The aligned file was used to assemble the transcripts, estimate their abundance, and detect the differential expression of genes. Differentially expressed genes were determined based on unique counts and multiple alignments using Bedtools [25]. The RT (read count) data were processed based on the quantile-quantile normalization method using EdgeR within R software [26] using Bioconductor [27]. Gene classification was based on searches conducted in the DAVID (http://david.abcc.ncifcrf.gov/) and Medline databases (http://www.ncbi.nlm.nih.gov/).

### 2.13. Real-Time Polymerase Chain Reaction

Real-time polymerase chain reaction (PCR) was performed using the StepONEPlus system (Applied Biosystems, CA, USA) with 2x power SYBR green master mix (AB, USA) under the following three-stage program parameters: 95°C for 10 min, 95°C for 15 sec, and 59°C for 30 sec (40 cycles). Primers for *ALU* (human) and *TGF-β* (human) were purchased from Bioneer Corporation (Daejeon, Korea). All PCR reactions were performed in triplicate. The comparative quantification of each target gene was performed based on the cycle threshold (*C*_*T*_), which was normalized to human *GAPDH* using the *ΔΔ*CT method proposed by Livak and Schmittgen.

### 2.14. Statistical Analyses

All values are presented as the mean ± standard error of the mean (S.E.M). One-way ANOVA was used to assess significance, and a *p* value of ≤0.05 was considered statistically significant. IBM SPSS software version 21.0 was used for all analyses.

## 3. Results

### 3.1. Primed MSCs Show Antiapoptotic Effects in the H4 Swedish Cell Line under Serum Starvation

**Figure 1:**
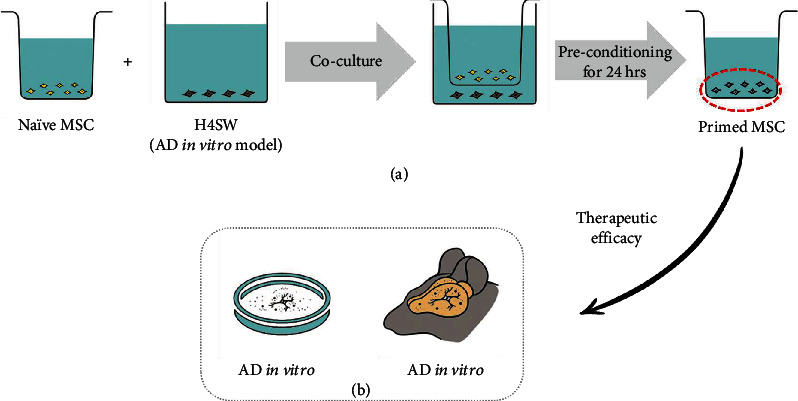
The concept of primed MSCs. (a) The procedure for preconditioning WJ-MSCs with H4SW cells to make primed MSCs. (b) After preconditioning, the therapeutic efficacy of primed MSC was evaluated in vitro and in vivo.

**Figure 2:**
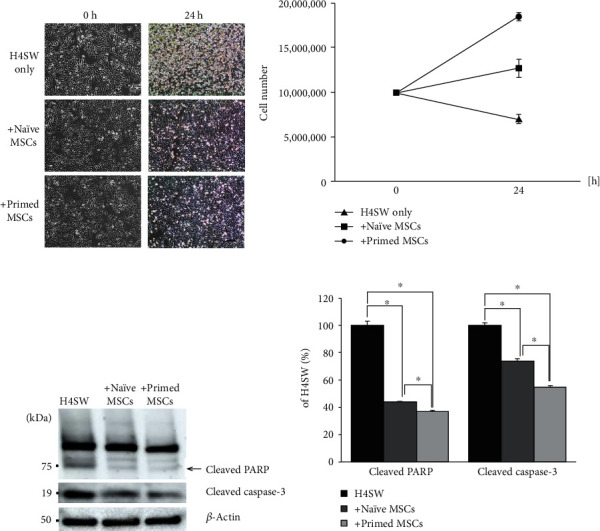
Antiapoptotic effect of primed MSCs. (a) Images of serum-starved H4SW cells cocultured with naïve MSCs or primed MSCs for 24 h. Scale bar: 40 *μ*m. (b) The number of viable cells in different experimental groups was counted by the trypan blue staining method. (c) Western blot analysis of cell death markers, cleaved PARP, and cleaved caspase-3. (d) The densitometry results are presented as fold change compared to H4SW cells. The data were normalized to *β*-actin expression. The data are presented as the mean ± S.E.M. Three samples per experimental group were tested in each assay. ^∗^*p* < 0.05.

To evaluate the therapeutic efficacy of primed MSCs, H4 Swedish cells (H4SWs) were cocultured with primed MSCs for 24 h (Figure 1(a)). Apoptosis was observed when the H4SW cells were in the serum-starvation state for 24 h (H4SW only). However, when naïve MSCs or primed MSCs were cocultured with H4SW cells, cell death was inhibited (Figure 2(a)). Following coculture, the number of viable cells was counted. Compared to the H4SW cell-only group, more viable cells were observed in the +naïve MSC and +primed MSC groups (Figure 2(b)). The antiapoptotic effects in the H4SW cell model were the highest in the +primed MSC group. Next, Western blot analysis was performed to confirm the antiapoptotic effect of naïve and primed MSCs. The expression of cell death markers, cleaved PARP, and cleaved caspase-3 was decreased when H4SW cells were cocultured with naïve MSCs and primed MSCs (Figures 2(c) and 2(d)). Based on the densitometric analysis, the levels of cleaved PARP and caspase-3 were significantly decreased in the +naïve MSC (2.2- and 1.4-fold changes, respectively) and +primed MSC groups (2.7- and 1.8-fold changes, respectively). From these results, we confirmed that primed MSCs exhibited stronger antiapoptotic effects on H4SW AD cells than naïve MSCs in the *in vitro* model.

### 3.2. Primed MSCs Show *In Vitro* Therapeutic Effects against Alzheimer's Disease

Next, we performed Western blot analysis to confirm the therapeutic efficacy of primed MSCs on AD pathology, especially A*β* and ubiquitin conjugates. A*β* is the most well-known pathological hallmark of AD. Ubiquitin conjugates are negatively correlated with 26S proteasome activity, which means that impaired 26S proteasome activity results in the accumulation of A*β*, hyperphosphorylated tau, and ubiquitin conjugates in the AD brain. Therefore, along with A*β*, the level of ubiquitin conjugates was measured in this study as another hallmark of AD.

**Figure 3:**
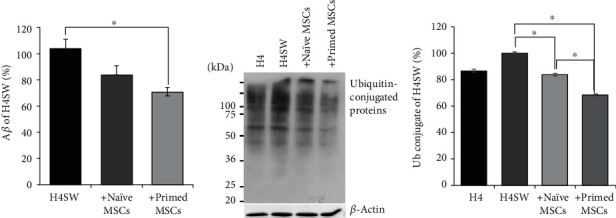
Therapeutic effect on Alzheimer's disease in vitro. (a) Secreted amyloid-beta levels in the conditioned media were measured by ELISA. ^∗^*p* < 0.05. (b) The degradation of ubiquitin (Ub) conjugates in H4SW cells with cocultured MSCs was measured by Western blots. The percentage of Ub conjugates was calculated as a percentage of that in the H4SW cells. (c) Changes in mRNA expression were evaluated. The data are presented as the mean ± S.E.M. Three samples per experimental group were tested in each assay. ^∗^*p* < 0.05.

Primed MSCs were cocultured with H4SWs *in vitro* for 24 h to evaluate the therapeutic efficacy against AD symptoms (Figure 3). Following coculture, the level of A*β* in the conditioned media was measured by ELISA (Figure 3(a)). Secreted A*β* was significantly reduced under the +primed MSC condition compared to the control H4SW cells (1.6-fold change). However, naïve MSCs did not show a statistically significant anti-A*β* effect. Next, the cumulative changes in the levels of ubiquitin conjugates were analyzed by Western blots (Figure 3(b)) and the intensity of the bands was quantified (Figure 3(c)). In the AD *in vitro* model (H4SW cells), more ubiquitin conjugates accumulated in the cytosol than in the normal cell line (H4). However, the level of ubiquitin conjugates was significantly attenuated in both +naïve MSCs (1.2-fold change) and +primed MSCs (1.4-fold change). In particular, primed MSCs showed enhanced therapeutic effects by attenuating ubiquitin conjugate accumulation. This demonstrated that primed MSCs successfully reduced the level of A*β* and ubiquitin conjugates in the AD *in vitro* model and that this effect was better than that of naïve MSCs.

In addition, the differences in gene expression (*APPSW*, *BACE1*, and *IGFBP3*) in H4 and H4SW cells cocultured with naïve MSCs or primed MSCs were analyzed. The analysis revealed that the dysregulated genes in the H4SW AD *in vitro* model were altered toward normal conditions (H4 cells) after coculture with naïve MSCs and primed MSCs. Between the two MSCs, primed MSCs showed better alteration (Supplementary Figure 1).

### 3.3. Evaluation of Therapeutic Efficacy of Primed MSCs in 5xFAD Mice

**Figure 4:**
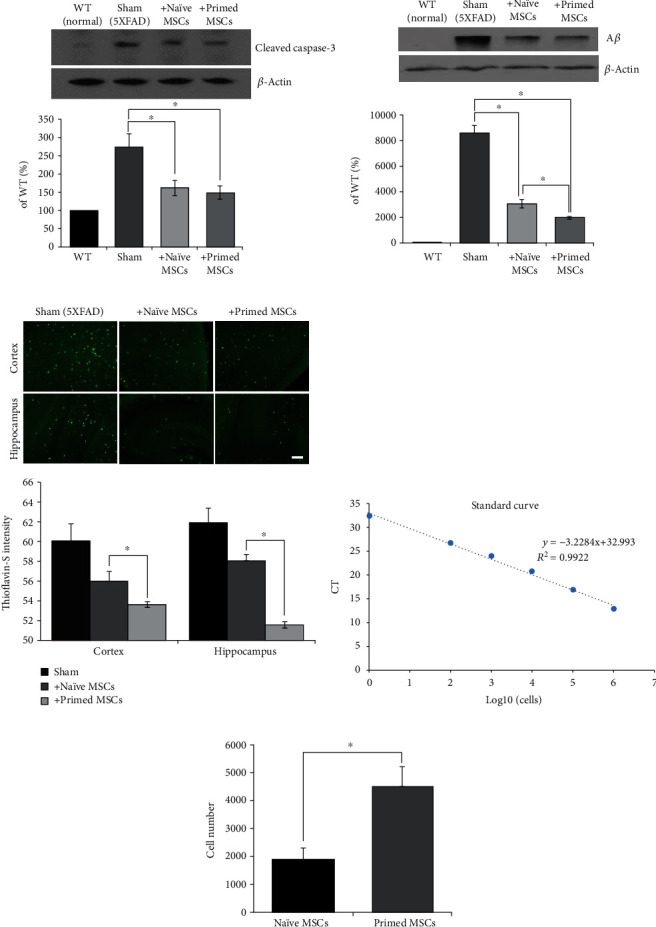
The therapeutic efficacy of primed MSCs in vivo. (a) The level of apoptosis was measured after the injection of naïve and primed MSCs into the lateral ventricle of mouse brains and expressed as a percentage of the WT controls. (b) Secreted amyloid-beta levels were measured by Western blots and expressed as a percentage of the WT controls. (c) The deposition of amyloid-beta in the cortex and hippocampus was visualized by thioflavin-S staining, and the intensity was measured and plotted as a histogram. Scale bar: 100 *μ*m. (d) The standard curve was fitted to the linear regression for real-time quantitative PCR (qPCR) analysis. (e) The absolute number of cells in the brains of mice in the naïve MSC and primed MSC groups was calculated. The data are presented as the mean ± S.E.M. *N* = 3 per group. ^∗^*p* < 0.05.

To evaluate the efficacy of primed MSCs in AD, we performed an *in vivo* experiment using a 5xFAD AD transgenic mouse. The experimental animals (12 months old) were divided into four groups: wild-type control (WT), transgenic control (sham), naïve-MSC, and primed MSC. We injected 1 × 10^5^ WJ-MSCs into the right lateral ventricle. One week after injection, the mice were euthanized, and brain tissues were harvested. First, the antiapoptotic effect of primed MSCs was assessed by cleaved caspase-3 Western blot analysis (Figure 4(a)). When compared to the WT mice, the 5xFAD mice showed increases in cleaved caspase-3, indicating neuronal death in the brain, whereas both the naïve MSCs and primed MSCs significantly reduced cleaved caspase-3 levels in the brain. Next, A*β* accumulation in the brain was measured by Western blots and thioflavin-S staining (Figure 4(b)). Compared to the WT control, the deposition of A*β* in the brain was observed in 5xFAD mice. The groups injected with naïve MSCs and primed MSCs showed decreases in A*β* accumulation. Primed MSCs, in particular, attenuated A*β* accumulation more effectively than naïve MSCs, which was confirmed by thioflavin-S staining. Thioflavin-S staining (Figure 4(c)) revealed extensive A*β* (green) deposits in the cortex and hippocampal regions of the 5xFAD transgenic mouse control group (sham). Strikingly, the amount of A*β* in the cortex and hippocampus was reduced in the groups injected with naïve and primed MSCs, and the primed MSC group showed better therapeutic efficacy.

Then, we quantified the number of naïve MSCs or primed MSCs in the 5xFAD brains via real-time quantitative PCR analysis using a human-specific *ALU* primer (Figure 4(e)). The absolute number of MSCs was determined based on the standard curve (linear regression *R*^2^ = 0.992, Figure 4(d)). Approximately 2,000 remaining cells were found in the mice injected with naïve MSCs, whereas increased numbers of primed MSCs were detected (2.4-fold change). Based on these results, the primed MSCs showed both enhanced therapeutic effects and increased cell survival *in vivo*.

### 3.4. Primed MSCs Differ from Naïve MSCs in mRNA Expression

**Figure 5:**
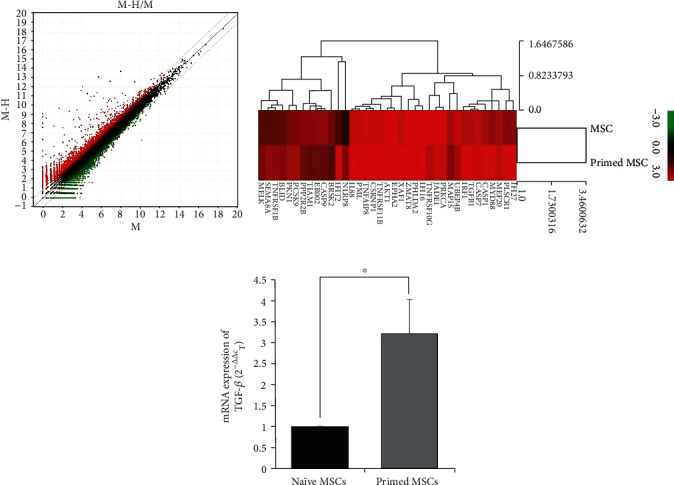
The mRNA expression patterns of primed MSCs. (a) Upregulated genes are represented in red and downregulated genes in blue. (b) Euclidean distance clustering of significant genes. (c) TGF-*β* expression in the naïve and primed MSCs analyzed by qPCR. The data are presented as the mean ± S.E.M. Three samples per experimental group were tested in each assay. ^∗^*p* < 0.05.

An RNA microarray was performed to identify the changes in mRNA expression in the primed MSCs (Figure 5), and a scatterplot was derived from the raw data (Figure 5(a)). In Figure 5(a), the upregulated genes in the primed MSCs were compared to the naïve MSCs and are shown in red and the downregulated genes are shown in blue. The Euclidean distance clustering of the significant genes analyzed by MeV software is presented as log-transformed data in Figure 5(b). The 38 upregulated genes were clustered as upregulated. Among these genes, we screened TGF-*β*, whose expression was increased over 3.0-fold in primed MSCs compared to the levels in naïve MSCs. Furthermore, the upregulation of TGF-*β* expression in primed MSC was confirmed via quantitative real-time PCR. The results showed that the primed MSCs expressed TGF-*β* at levels 3.2-fold higher than those in naïve MSCs (Figure 5(c)).

These results demonstrated that TGF-*β*, which is highly secreted by primed MSC, could be a key molecule for therapeutic efficacy on AD.

### 3.5. Therapeutic Efficacy of TGF-*β* in 5xFAD Mice

**Figure 6:**
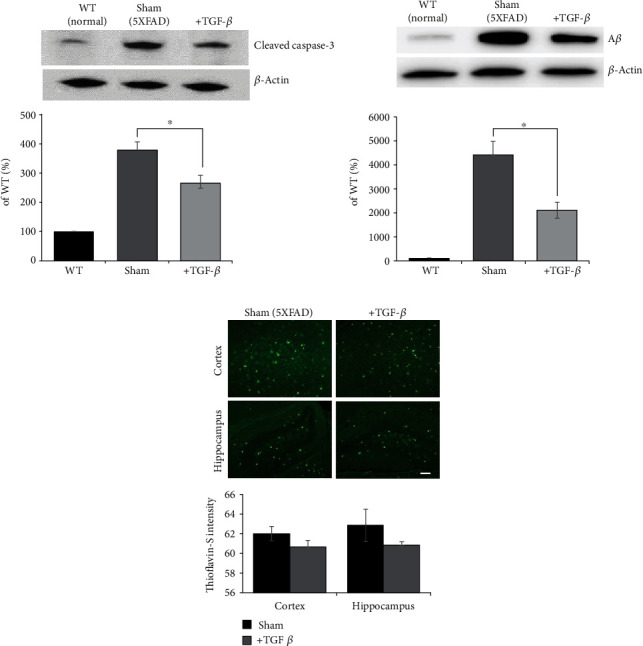
Therapeutic efficacy of TGF-*β* in 5xFAD mice. (a) Changes in cleaved caspase-3 were measured in the experimental groups: WT, sham, and TGF-*β*. (b) Secreted amyloid-beta levels in the brain were measured. (c) Thioflavin-S staining was performed, and fluorescent images from the cortex and hippocampus of each group are shown. Scale bar: 100 *μ*m. The data are presented as the mean ± S.E.M. ^∗^*p* < 0.05.

To determine the role of TGF-*β*, especially in antiapoptosis and anti-A*β*, recombinant protein was injected into the lateral ventricle of 5xFAD mice followed by euthanasia one-week later (Figure 6). Western blot analysis revealed that cleaved caspase-3 was increased in the 5xFAD mice compared to the WT controls but was significantly decreased in the TGF-*β* group (Figure 6(a)). Next, the anti-A*β* effect of TGF-*β* was measured by A*β* Western blot analysis and thioflavin-S staining (Figures 6(b) and 6(c)). The deposition of A*β* in 5xFAD mice was reduced following treatment with TGF-*β* (Figure 6(b)). However, this observation was not replicated, and no statistical significance was observed in the histological analysis of thioflavin-S staining (Figure 6(c)).

### 3.6. SRGN Secretion by H4SW Cells: A Potential Preconditioning Factor Inducing AD

**Figure 7:**
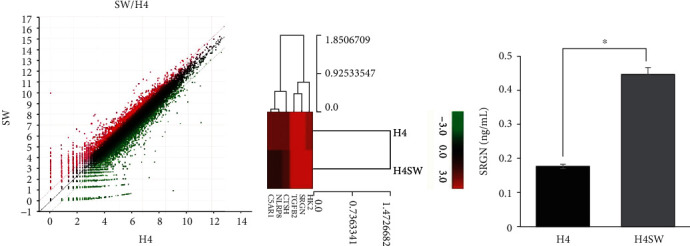
Identification of SGRN as a secretory protein of H4SW cells. The gene expression patterns of H4SW cells were upregulated compared to H4 cells. (a) Scatter plot and (b) Euclidean distance clustering of significant genes performed by MeV software are presented as log-transformed data. The green and red colors indicate decreased and increased gene expression, respectively. (c) ELISA assay of SRGN concentrations in H4 and H4SW cell cultures. Three samples per experimental group were tested in each assay. The data are presented as the mean ± S.E.M. ^∗^*p* < 0.05.

Next, which molecule caused primed MSCs to secrete TGF-*β* was investigated. To identify the potential candidates responsible for priming the MSCs, the gene expression profiles of H4 and H4SW cells were analyzed (Figure 7). The red dots in the figure denote increased mRNA expression of the H4SW cells compared to the H4 cells, and the green dots indicate decreased expression (Figure 7(a)). A total of six genes highly upregulated in the H4SW cells were selected, and clustering using the Euclidean distance measurements of significant genes was conducted (Figure 7(b)). Next, the amount of secreted serglycin (SGRN) proteins was measured in the conditioned media. In the H4SW cells, the level of SGRN protein was significantly elevated compared to the H4 cells, suggesting that the SGRN protein may represent the AD microenvironment and potentially act as the main inducer of primed MSCs.

### 3.7. SGRN Is the Main Effector of Primed MSCs

**Figure 8:**
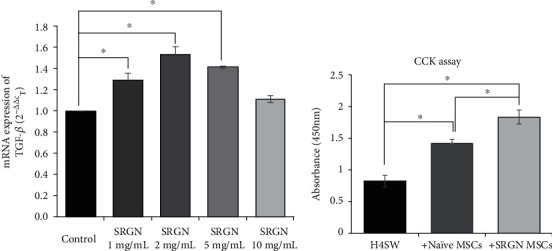
Effects of SRGN-primed MSCs. (a) The mRNA was extracted from SRGN-primed MSCs and analyzed by qPCR using a specific primer for human TGF-*β*. ^∗^*p* < 0.05, *n* = 3. (b) Viability of H4SW cells under serum-starved conditions analyzed by CCK-8 assays. Three samples per experimental group were tested in each assay. The data are presented as the mean ± S.E.M. ^∗^*p* < 0.05.

To confirm whether SGRN was the main inducer of primed MSCs, various concentrations of SGRN protein were used to treat naïve MSCs (Figure 8). After the treatment of the naïve MSCs with SGRN for 24 h, the TGF-*β* mRNA expression in naïve MSCs was measured via quantitative real-time PCR analysis. Significant increases in TGF-*β* mRNA expression were observed, except at 10 ng/mL (Figure 8(a)). The peak was observed at 2 mg/mL SGRN treatment. Next, the therapeutic potential of SGRN-treated MSCs (SGRN MSCs) was briefly assessed (Figure 8(b)). H4SW cells were cocultured with naïve MSCs or SRGN MSCs for 24 h, and then, the CCK assay was conducted to confirm the antiapoptotic effect of naïve and SRGN MSCs on H4SW cells. Cell death was significantly inhibited when H4SW cells were cocultured with naïve MSCs and SGRN MSCs. This suggests that SGRN secreted by H4SW cells or the AD microenvironment is an inducer of primed MSCs.

## 4. Discussion

Recent advances have demonstrated the promising therapeutic role of MSCs in AD [12, 17]. Because AD remains a major cause of morbidity and mortality, significant effort has been directed toward A*β* removal via stem cell transplantation [13, 28]. The therapeutic properties of MSCs are largely related to their antiapoptotic and anti-inflammatory abilities, which have been confirmed both *in vivo* and *in vitro* [13, 29, 30]. However, the low survival rates of MSCs *in vivo* are a challenge and the benefits of MSCs are mediated by undefined mechanisms [31–33].

Various modifications of MSCs have been attempted to improve their survival rates and therapeutic efficacy [31, 32, 34, 35]. Attempts to improve stem cell survival, metabolism, or migration ability have focused on genetic modifications to knock-out or knock-in specific genes [36–38]. However, the clinical application of genetically modified MSCs is associated with the risk of unexpected genetic mutations resulting in tumor formation [39]. In another approach, biocompatible scaffolds as an alternative to encapsulated MSCs have been developed to improve the survival and engraftment rates [40]. This method facilitated clinical application but did not improve the efficacy of MSCs.

In recent years, preconditioning methods that attempted to improve the efficacy of MSCs have also been in the spotlight [41–43]. Preconditioning aims to promote cell proliferation [43], improve migratory ability [43], and enhance protein secretion [44]. Unlike genetic modifications, preconditioning can be achieved by exposing MSCs to specific microenvironments. Compared with genetic modifications, preconditioning enhanced therapeutic efficacy while maintaining the genotype of the cells [45]. A number of 0approaches have been proposed to make preconditioned MSCs. Preconditioning by hypoxia [46], inflammatory stimuli [42, 45], or other factors [42] are strategies designed to enhance the survival and effectiveness of MSC posttransplantation. In this study, we preconditioned MSCs using A*β*, the most important hallmark of Alzheimer's disease, and used H4SW cells for preconditioning through endogenous A*β*.

H4SW cells are a stable cell line whereby the amyloid precursor protein (APP) Swedish mutation was introduced into a human glioblastoma cell line. APP is an integral membrane protein of neuronal cells involved in synaptic formation, synaptic plasticity, and ion export. APP, expressed in cell membranes, is usually cleaved by *α*-secretase. However, mutations in APP protein or PSEN1/PSEN2 increase the change for APP to be cleaved by *β*- and *γ*-secretase, resulting in high levels of A*β* production in the brain. The A*β* produced is considered the causative substance of Alzheimer's disease, as it forms oligomer aggregates and A*β* plaques, resulting in neuronal toxicity and ultimately, the death of neuronal cells. APP Swedish, which is adjacent to the *β*-secretase site in APP, is one of the well-known genetic mutations in familial Alzheimer's disease, resulting in increased total A*β* production [47–49]. Therefore, the research model for AD with an APP Swedish mutation is now widely used [50–53], and the H4SW cell line is called the AD *in vitro* model [20]. Moreover, A*β* accumulated in the brain of AD patients activates glial cells, which are known to eliminate A*β* and have neuroprotective effects [54–57]. MSCs do not remove A*β* itself when exposed to AD but secrete proteins that can stimulate neurons or glial cells through paracrine action [13]. Therefore, we propose that the H4SW cell line was suitable for this study because the therapeutic efficacy of MSCs can be evaluated by measuring the reduction in A*β* deposits by stimulated H4SW cells. In addition, the deposition of ubiquitin conjugates, an indirect marker for ubiquitin proteasome activity which is closely related to the amyloid-beta clearance mechanism, was also measured [15, 58].

When H4SW cells were cocultured with primed MSCs, decreases in the level of A*β* and ubiquitin conjugates were observed in the H4SW cells (Figures 2 and 3). In addition, when primed MSCs were administered directly into the brain of 5xFAD mice, an AD *in vivo* model, primed MSCs showed the therapeutic effects of suppressing neuronal death and promoting A*β* clearance (Figure 4). Messenger RNA sequencing confirmed that SGRN secreted by H4SW cells promoted TGF-*β* secretion by MSCs (Figures 5 and 7). TGF-*β* protein had the same anticell death and anti-A*β* effects as primed MSCs, and SRGN-treated MSCs showed anticell death effects (Figures 6 and 8). Moreover, both TGF-*β* protein and primed MSCs showed an anti-inflammation effect resulting in inducing a decrease in easing the level of TNF-*α* and GFAP as a common marker for neuroinflammation in 5xFAD (Supplementary Figure 4). It is known that the secretion of SRGN is increased when an inflammatory reaction occurs [59]. Heparin sulfate proteoglycan, which contains SRGN, was responsible for promoting the fibrillization of A*β* and tau proteins [60]. Interestingly, it was also reported that *SRGN* gene expression and protein expression were significantly increased in AD patients compared to normal controls [61]. Thus, SRGN may be thought of as a possible biomarker for AD, suggesting that the preconditioning of SRGN in MSCs may be possible to generate enhance MSC for AD treatment. Additionally, TGF-*β* is highly expressed in primed MSCs or SRGN-treated MSCs, the signaling pathway associated with TGF-*β* is impaired in AD [62], and TGF-*β* itself showed neuroprotective effects [63, 64]. Thus, the prior findings that SRGN and TGF-*β* are related to neuroinflammation in the AD patients' brain have been confirmed once aging through this study. Therefore, we concluded that TGF-*β*, highly secreted by primed MSCs, can have therapeutic efficacy in AD.

A particularly noteworthy finding is that when MSCs were exposed to an AD microenvironment, SRGN secreted locally in the Alzheimer's brain was recognized by the MSCs, which were induced to increase the expression of TGF-*β*, promoting therapeutic efficacy. As far as we know, this is the first study to generate preconditioned MSC using a possible biomarker for the target disease. Like the concept of vaccination, we can make MSCs in a ready-to-fight state, promoting the secretion of effective proteins by exposing them to the target disease microenvironment in advance.

Our study had several limitations. First of all, the exact mechanism of action of SGRN, MSC, and TGF-*β* was not elucidated. Second, the recovery of cognition in the AD *in vivo* model was not studied. After the injection of primed MSCs, TGF-*β*, or SRGN MSCs, a long-term follow-up must be observed. Finally, the optimization of signaling factors and their combinations used in MSC preconditioning requires further investigation. Studies based on preconditioned MSCs should be conducted to enhance the therapeutic capacity of MSCs and expand the platform developed in this study.

## 5. Conclusions

In summary, we report that AD environmental preconditioning is a promising strategy to reduce cell death and ubiquitin levels while maintaining the stemness and characteristics of MSCs. Further, these data suggest that human WJ-MSCs exposed to an AD cell model *in vitro* may represent a promising and novel therapy for AD.

## Data Availability

Data can be available on request.

## Abbreviations

AD: Alzheimer's disease
MSCs: Mesenchymal stem cells
WJ-MSCs: Wharton's jelly-derived mesenchymal stem cells
NEP: Neprilysin
A*β*: Amyloid-beta
BACE1: Beta-secretase 1
H4SW: APP695-Swedish mutant (K595N/M596L)-expressing H4
CSF: Cerebrospinal fluid
Ub: Ubiquitin
GFAP: Glial fibrillary acidic protein
TNF-*α*: Tumor necrosis factor-alpha
TGF-*β*: Transforming growth factor-beta
SRGN: Serglycin
MEM: Minimum essential media
PCR: Polymerase chain reaction
ELISA: Enzyme-linked immunosorbent assay
GMP: Good manufacturing practice
FBS: Fetal bovine serum
PBS: Phosphate-buffered saline
ISCT: International Society for Cell and Gene Therapy
S.E.M.: Standard error of the mean
ALU: Arthrobacter luteus element
ANOVA: Analysis of variance.

## Supplementary Materials

Supplementary 1 Supplementary Figure 1: changes in mRNA expression of H4SW cells cocultured with naïve MSCs or primed MSCs. Changes in mRNA expression of H4SW cells when cocultured with naïve MSCs and primed MSCs. The data are presented as the mean ± S.E.M.

Supplementary 2 Supplementary Figure 2: validation of stemness in primed MSCs. The surface markers of primed MSCs were assessed by flow cytometry, and the panels were constructed according to the ISCT MSC criteria.

Supplementary 3 Supplementary Figure 3: validation of stemness following SGRN treatment of MSCs. MSC surface markers after SGRN treatment were assessed by flow cytometry, and the panels were constructed according to the ISCT criteria.

Supplementary 4 Supplementary Figure 4: anti-inflammatory efficacy of primed MSCs and TGF-*β* in 5xFAD mice. (A) Western blot analysis of inflammatory markers, GFAP and TNF-*α*. (B) The densitometry results are presented as fold change compared to WT controls. The data were normalized to *β*-actin expression. The data are presented as the mean ± S.E.M. Three samples per experimental group were tested in each assay. ^∗^*p* < 0.05.
