# Supplementary figures and images for: Exposure of Mesenchymal Stem Cells to an Alzheimer's Disease Environment Enhances Therapeutic Effects
Source: Stem Cells Int. 2021 Mar 16;2021:6660186. doi: 10.1155/2021/6660186 (PMC7988745; doi:10.1155/2021/6660186)

**(A)**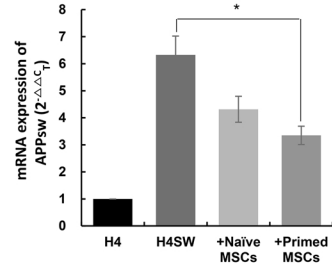**(B)**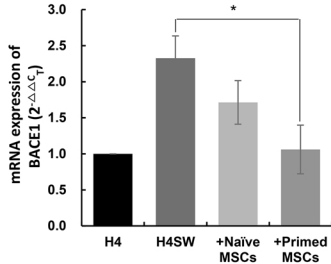**(C)**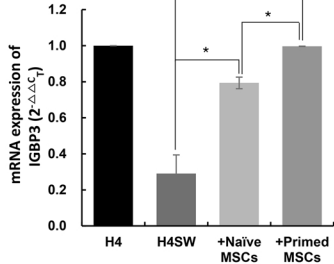

Supplement: Supplementary 1 — Supplementary Figure 1: changes in mRNA expression of H4SW cells cocultured with naïve MSCs or primed MSCs. Changes in mRNA expression of H4SW cells when cocultured with naïve MSCs and primed MSCs. The data are presented as the mean ± S.E.M. [file 6660186.f1.pdf]

**(A)**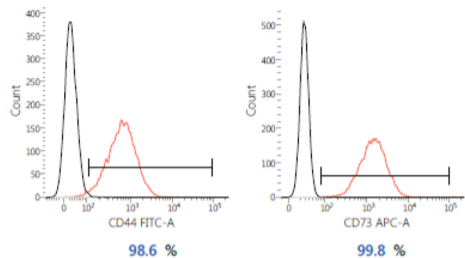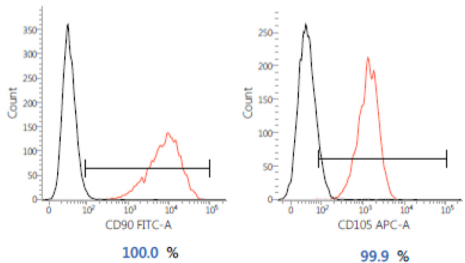**(B)**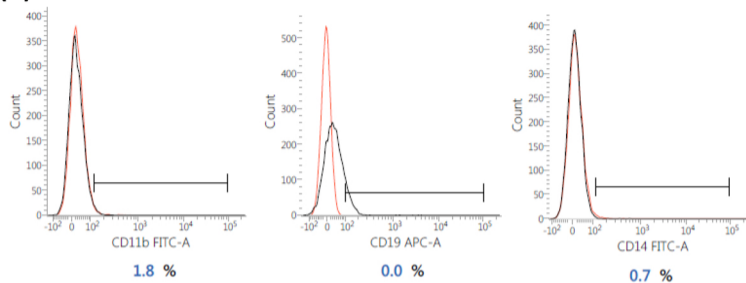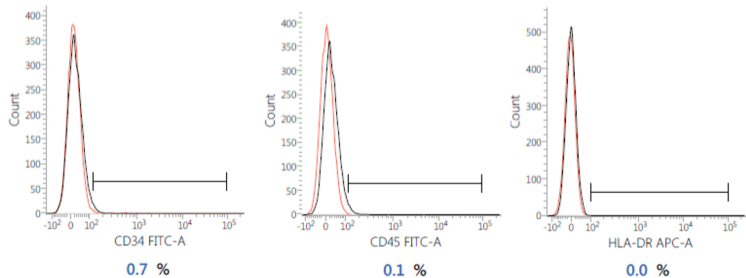

Supplement: Supplementary 2 — Supplementary Figure 2: validation of stemness in primed MSCs. The surface markers of primed MSCs were assessed by flow cytometry, and the panels were constructed according to the ISCT MSC criteria. [file 6660186.f2.pdf]

**(A)**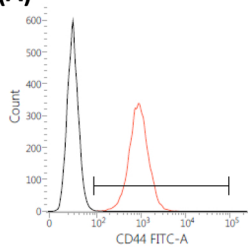**99.9 %**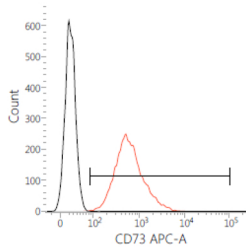**99.9 %**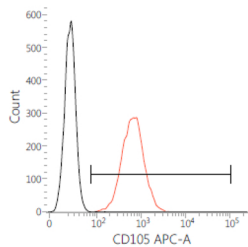**100.0 %**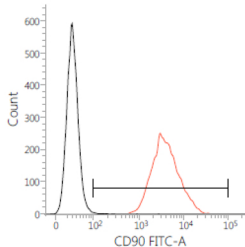**100.0 %****(B)**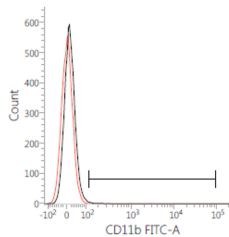**0.0 %**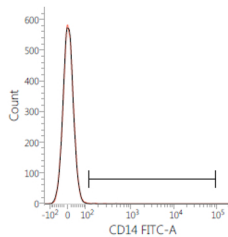**0.1 %**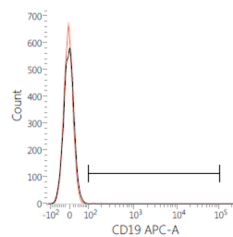**0.0 %**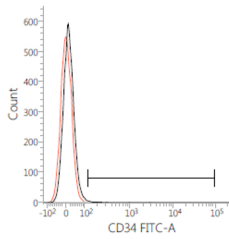**0.1 %**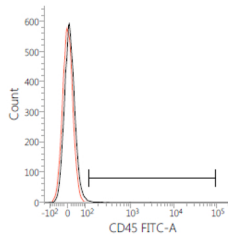**0.0 %**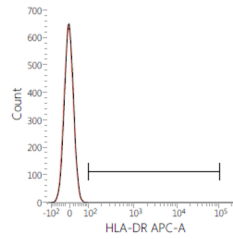**0.0 %**

Supplement: Supplementary 3 — Supplementary Figure 3: validation of stemness following SGRN treatment of MSCs. MSC surface markers after SGRN treatment were assessed by flow cytometry, and the panels were constructed according to the ISCT criteria. [file 6660186.f3.pdf]

**(A)**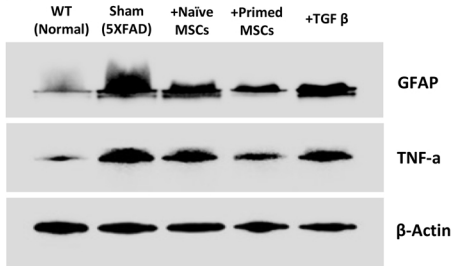**(B)**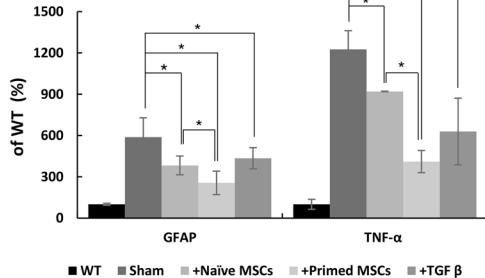

Supplement: Supplementary 4 — Supplementary Figure 4: anti-inflammatory efficacy of primed MSCs and TGF-β in 5xFAD mice. (A) Western blot analysis of inflammatory markers, GFAP and TNF-α. (B) The densitometry results are presented as fold change compared to WT controls. The data were normalized to β-actin expression. The data are presented as the mean ± S.E.M. Three samples per experimental group were tested in each assay. ∗p < 0.05. [file 6660186.f4.pdf]
